# Directing the Future Breakthroughs in Immunotherapy: The Importance of a Holistic Approach to the Tumour Microenvironment

**DOI:** 10.3390/cancers13235911

**Published:** 2021-11-24

**Authors:** Hannah V. Newnes, Jesse D. Armitage, Katherine M. Audsley, Anthony Bosco, Jason Waithman

**Affiliations:** Telethon Kids Institute, The University of Western Australia, Nedlands, WA 6009, Australia; hannah.newnes@telethonkids.org.au (H.V.N.); jesse.armitage@telethonkids.org.au (J.D.A.); katherine.audsley@telethonkids.org.au (K.M.A.); anthony.bosco@telethonkids.org.au (A.B.)

**Keywords:** tumour microenvironment, immunotherapy, multi-omics, personalised therapy

## Abstract

**Simple Summary:**

Immunotherapies have changed the way we treat cancer and, while some patients have benefitted greatly, there are still those that do not respond to therapy. Understanding why some patients respond to therapy and others do not is critical in developing new immunotherapeutic strategies. The increasing awareness of the importance of investigating the tumour in its entirety, including the surrounding tissue and role of various immune cells is helping to differentiate responders and non-responders. In addition, the resolution gained by the development of sophisticated bioinformatic technologies allows for a deeper understanding of the complex roles of individual cells in the tumour. This advancement will be critical for the development of novel therapies to treat cancer.

**Abstract:**

Immunotherapy has revolutionised the treatment of cancers by exploiting the immune system to eliminate tumour cells. Despite the impressive response in a proportion of patients, clinical benefit has been limited thus far. A significant focus to date has been the identification of specific markers associated with response to immunotherapy. Unfortunately, the heterogeneity between patients and cancer types means identifying markers of response to therapy is inherently complex. There is a growing appreciation for the role of the tumour microenvironment (TME) in directing response to immunotherapy. The TME is highly heterogeneous and contains immune, stromal, vascular and tumour cells that all communicate and interact with one another to form solid tumours. This review analyses major cell populations present within the TME with a focus on their diverse and often contradictory roles in cancer and how this informs our understanding of immunotherapy. Furthermore, we discuss the role of integrated omics in providing a comprehensive view of the TME and demonstrate the potential of leveraging multi-omics to decipher the underlying mechanisms of anti-tumour immunity for the development of novel immunotherapeutic strategies.

## 1. Introduction

It is an accepted view that solid cancers comprise not only malignant cells but a complex and dynamic network of tumour cells, immune cells, endothelial cells and vasculature, fibroblasts and an extracellular matrix containing various cytokines, chemokines, hormones, and growth factors. Additional factors including glucose levels, amino acids, pH, metabolites, and hypoxia all play an influential role in shaping the tumour microenvironment (TME) [1]. As such, understanding solid tumours requires a holistic approach that takes into consideration all these factors and the impact they have on cancer progression or control. Immune cells in the TME comprise both immunosuppressive cells that dampen immunity and effector cells associated with tumour clearance. The TME can often simply be characterised into two categories: (i) hot/T cell inflamed or (ii) cold/non-T cell inflamed, attributed to levels of pro-inflammatory cytokines and T cell infiltration [2]. Hot tumours in general are considered more responsive to immunotherapies than their cold counterparts. Focus to date has largely been limited to identifying strategies to convert cold tumours into hot tumours (reviewed elsewhere [3,4]). However, many immunotherapies have failed to reach the expected outcomes in the clinical setting, despite early promising results. In fact, characteristics of a hot tumour have been implicated in a failure to respond to checkpoint blockade and adoptive cellular therapies [5,6,7,8]. A binary approach does not consider all the nuances of the TME, or the promiscuity and plasticity of the cells within. Such environments are constantly evolving under pressure from the immune system and the continuous growth of the cancer itself. Generating a deeper understanding of the TME is critical to developing strategies to induce responses in all patients, particularly those who currently do not respond to therapy despite having a ‘hot’ tumour.

## 2. Overview of Immunotherapy

The immune system can prevent progression of neoplastic cells into palpable tumours by engaging in a process known as cancer immunoediting [9,10]. During this process, immune cells abolish tumour growth by purging neoplastic cells in a process termed ‘elimination’. Alternatively, cancer outgrowth is suppressed by establishing a state of tumour-immune ‘equilibrium’. However, when disease manifests clinically, the cancer cells ‘escape’ immune-mediated control and immune pressure selects for variants resistant to immune detection [10]. The goal of immunotherapy is to overcome or mitigate tumour-induced immunosuppression and enable immune-mediated tumour clearance.

Immunotherapies have revolutionised the field of cancer treatment, with the advances in immune checkpoint blockade (ICB) and adoptive cell therapy (ACT) increasingly becoming the standard of care across a growing number of malignancies. ICB utilises antibodies to prevent receptor–ligand binding of inhibitory signals on the immune system [11]. FDA-approved ICB include anti-PD-1/PD-L1, which block signals inhibiting T cell effector function [12], and anti-CTLA4, which blocks inhibitory signals during interactions between antigen presenting cells and T cells to promote effective priming [13]. ACT describes autologous tumour-specific T cell transfer into patients to eradicate cancer cells, either in the form of endogenous T cells [14] with tumour specificity or engineered T cells with a chimeric antigen receptor (CAR) or T cell receptor (TCR) [15]. Most immunotherapies focus on boosting the anti-tumour CD8^+^ T cell response to generate a therapeutic effect. Despite ongoing efforts to extend the therapeutic potential of these immunotherapies, particularly focusing on combinatorial approaches with traditional treatments and other immune-based therapies, there are still significant limitations in the clinical efficacy of these treatments with a large proportion of patients not responding to therapy [11,16].

One barrier to successful immunotherapies is the quality of the ensuing immune response. A significant focus to date has been on identifying specific markers associated with response to immunotherapy and treating patients that possess these ‘hot’ tumours enriched with cytotoxic T cells. However, data have emerged suggesting this is an oversimplified approach to a complex and dynamic interaction between immune cells and cancer cells. Unfortunately, CD8^+^ T cell infiltration is not always a direct correlate with response to ICB. Girlado et al. showed that neither CD8^+^ T cell density nor the juxtaposition of CD8^+^ cells to PD-L1^+^ cells correlated with response in Merkel cell carcinoma following treatment with pembrolizumab [17]. In addition, limitations of PD-L1 as a biomarker for response to ICB were highlighted in a recent review by Stern [18]. Hugo et al. analysed whole-exome sequences of 38 pre-treatment (pembrolizumab and nivolumab) melanoma tumours and demonstrated that genes with putative roles in modulating the response to ICB were not differentially expressed between responding and non-responding tumours [5]. Furthermore, T cell-related genes such as CD8A/B, PD-L1, LAG3 and IFNG were not more highly expressed in anti-PD-1-responsive tumours. In the TME, CD8^+^ T cells often express multiple inhibitory receptors (LAG3, TIGIT, TIM3, NKG2A) in addition to PD-1; these cells are resistant to activation, have a reduction in proliferative capacity, cytokine production and cytotoxic ability, and possess markers associated with apoptosis [19]. These tumour-infiltrating T cells are dysfunctional, and a single agent checkpoint blockade alone will not be sufficient. In fact, a compensatory mechanism has been proposed, whereby anti-PD-1 treatment led to increased expression of LAG3 and TIM3 [20]. As such, clinical trials are currently underway testing bispecific humanised antibodies for LAG3 and PD-1 (NCT03219268), LAG3 and PD-L1 (NCT03440437) and TIM3 and PD-1 (NCT03708328), as well as combinatorial treatments of anti-PD-1/L1 and anti-TIM3 monoclonal antibodies (NCT02608268, NCT02817633, NCT03744468). Promisingly, interim results from the RELATIVITY-047 clinical trial combining anti-LAG3 and anti-PD-1 in advanced melanoma showed significant increase in progression free survival compared to anti-PD-1 alone (NCT03470922) [21]. The success of these approaches highlights how improving our understanding of tumour-infiltrating cells can inform on improved strategies of targeting them.

In recent years, there has been an increasing focus on the TME as a whole in solid tumours and the role individual components play in modulating response to immunotherapies. Herein, this review analyses major cell populations present within the TME with a focus on their diverse and often contradictory roles in cancer and how this informs our understanding of immunotherapy.

## 3. Cell Types

### 3.1. Cancer-Associated Fibroblasts

Cancer-associated fibroblasts (CAFs) are a key cell types in the TME and are critical in regulating both tumour and immune cells. They are a heterogeneous group of tumour-infiltrating activated fibroblasts with a mesenchymal cell lineage. CAFs are defined by biomarkers including but not limited to fibroblast activation protein (FAP), and platelet-derived growth factor alpha (PDGFα) and alpha-smooth muscle actin (αSMA) [22]. Fibroblasts differentiate into CAFs through secretion of growth factors, transcription factors, metalloproteinases (MMPs), cytokines, and reactive oxygen species (ROS) by tumour cells and/or immune cells [22,23]. CAFs themselves are major sources of growth factors, cytokines, chemokines, extracellular vesicles, extracellular matrix, proteins, and various enzymes [23]. In turn these factors can affect tumour initiation, progression, and therapeutic resistance.

Although CAFs have received considerable attention in the literature, numerous questions remained unanswered. In particular, the crosstalk between CAFs and immune cells needs more in-depth research and analysis. The focus so far has been largely limited to the tumour-promoting function of CAFs; however, tumour-suppressive functions have been identified, particularly in the early stages of tumour development. For example, it has been found that subpopulations of CAFs activated by matrix-specific Hedgehog inhibit tumour growth and progression in multiple animal tumour models, including bladder, colon, and pancreatic cancer [22,24]. Studies in pancreatic ductal adenocarcinoma (PDAC) have further challenged the notion of tumour-promoting CAFs by demonstrating increased tumour growth after depletion of αSMA^+^CAFs. PDAC lesions with depletion of αSMA-CAFs had an activated EMT signature with increased numbers of cancer stem cells and T_reg_ infiltration [25]. Moreover, Qian et al. demonstrated FAP^+^αSMA^+^CAFs fused with dendritic cells promoted CD8^+^ T cell activation in vitro and these activated T cells were able to inhibit tumour cell growth in vivo [26]. Finally, reduced numbers of CAFs in patients were correlated with decreased survival [25].

Single cell transcriptomics has identified multiple previously undetected subsets of CAFs and been instrumental in demonstrating their complex role in the TME. A comparison between stromal cells from human lung tumours and matching non-malignant lung samples revealed the identity of multiple subtypes of CAFs, all expressing a unique set of proteins that differentiated them from non-malignant fibroblasts [27,28]. Interestingly, Elyada et al. employed single cell RNA sequencing (scRNAseq) to interrogate CAF heterogeneity in PDAC samples and identified a novel population of CAFs with high expression of MHC-II [29], demonstrating their ability to act as antigen presenting cells (APCs) and present antigens to CD4^+^ T cells. In summary, increasing evidence strongly suggests that CAFs have diverse functions, implying that both pro-tumoral and anti-tumoral CAFs coexist in the stroma [24,30]. More thorough research is needed to fully comprehend the interactions between CAFs, tumour cells and immune cells in the TME. Elucidating the role of CAFs in modulating the TME, particularly with the use of next generation technologies, is expected to deepen our understanding of tumour-evolution mechanisms and tumour immunotherapy.

### 3.2. Tumour Endothelial Cells

In healthy tissue, endothelial cells (ECs) are typically quiescent due to a finely tuned balance of angiostatic and angiogenic factors. However, ECs become activated under environmental stressors by sensing a gradient of pro-angiogenic signals to invade surrounding stroma and generate new blood vessels to increase immune cell trafficking to the area of inflammation. As cancer cells become hypoxic, they induce an ‘angiogenic switch’ leading to expression of angiogenic factors including HIF1a, vascular endothelial growth factor A (VEGFA), PDGF, angiopoietin-2, pro-angiogenic chemokines and receptors [31]. This results in chronically activated ECs, termed tumour endothelial cells (TECs), that induce continuous propagation of new blood vessels to support tumour development [32].

TECs are a multifaceted population with a known role in promoting tumour angiogenesis. However, TECS also act as significant mediators of immune regulation. They induce a process referred to as ‘endothelial anergy’ characterised by insensitivity to inflammatory cues and abnormal leukocyte–vessel interactions [33]. Yet, TECs are associated with T cell priming, activation, and proliferation by acting as APCs. Key features of TECs include their high proliferative potential and critically altered gene expression including pro-angiogenic factors and stemness genes [31], causing enhanced secretion of immunomodulatory cytokines and altered expression of receptors. Interestingly, STING has shown to be highly expressed by the ECs of high endothelial venules (HEV). STING activation has been implicated in tumour vessel maturation and inhibition of vessel propagation, a critical factor in tumour angiogenesis [34]. However, STING activation can also enhance the upregulation of adhesion molecules on ECs and induces infiltration of CD8^+^ T cell into the TME promoting an anti-cancer immune responses [31]. Similarly, naïve lymphoid endothelial cells (LECs) of extra-tumoral lymph nodes are known to attract and cross-prime naïve CD8^+^ T cells by acting as semi-professional APCs [35]. Vokali et al. showed that LECs could generate antigen-experienced T cells with memory-like functions that rapidly evolve effector functions upon pro-inflammatory stimulation, suggesting these T cells would be preferable for anti-tumour response [36].

Subpopulations of TECs also contribute to the formation of tertiary lymphoid structures (TLS), which can drive an effective anti-tumour response [37]. In cancer, TLS represent a crucial site for antigen presentation by DCs and the proliferation of T and B cells [31,38,39]. Importantly TLS density is predictive of response to anti-PD-1/PD-L1 therapy, but the putative mechanisms remain poorly understood. Future work, particularly the use of next generation sequencing technologies, to understand this phenomenon could provide opportunities to improve response to ICB. Clinically, evidence suggests that the presence of HEV acts as a favourable prognostic factor in melanoma, pancreatic cancer, non-small cell lung carcinoma (NSCLC) and colorectal cancer (CRC) [39,40,41,42].

In contrast, T cell inhibitory mechanisms of TECs involve Fas ligand (FasL), a homeostatic mediator of T cell apoptosis [31]. Preclinical data demonstrated that TECs expressing FasL were able to deplete CD8^+^ T cells in tumours while maintaining T_regs_. FasL expression was induced by tumour-derived VEGFA, IL-10 and prostaglandin E2 (PGE2) [43]. Importantly, TECs have been shown to express PD-L1, PD-L2 and TIM3 [31], thereby having the potential to directly inhibit T cell activation, while also providing the rationale that ICB could mitigate this effect.

### 3.3. Tumour-Associated Macrophages

Macrophages function to protect host cells from pathogens, and regulate cell turnover, tissue remodelling and wound repair. Macrophages are inherently plastic, and their activation is driven by environmental cues. Tissue resident macrophages and monocyte derived macrophages both contribute to tumour-associated macrophage (TAM) development [44]. Accumulating evidence demonstrates the critical role TAMs play in coordinating the pro- and anti-tumour effector mechanisms of the immune system in response to the TME.

Two subtypes of TAMs, M1 and M2 macrophages, have been described by their gene signatures in response to activation by type 1 (Th1) or type 2 (Th2) cytokines, respectively [45]. Accordingly, M1 and M2 macrophages are viewed as having contrasting functions where M1-like macrophages are pro-inflammatory, immunogenic, and anti-tumoral, whereas M2-like macrophages are anti-inflammatory, tolerogenic, angiogenic and pro-tumoral. Clinical studies have shown that TAM infiltration in solid tumours is associated with an M2 gene signature and worse outcomes [46]. However, other studies have proposed that macrophage infiltration may be associated with favourable outcomes for patients in prostate cancer [47], CRC [48] and NSCLC [49], although the mechanisms underlying this anti-tumour role remain unclear.

The M1/M2 paradigm is considered by many an oversimplification that does not reflect TAMs’ pro- and anti-inflammatory activities. A lack of sufficient markers, suitable mouse models, and in vitro systems has impeded the analysis of TAMs. Chevrier et al. identified, through transcriptomic analysis of human renal cell carcinoma tumour samples, 17 subgroups of TAMs with different gene expression profiles, involvement in immune suppression and influence on prognosis [50]. TAMs co-expressed pro-tumour markers CD204 and CD206, and anti-tumour markers CD169 and CD38, the latter a marker exclusively found upon M1 polarisation in murine macrophages [50]. Garrido-Martin et al. demonstrated that M2 and M1 signatures are not mutually exclusive within a single cell, demonstrating macrophages can have both M1-like and M2-like signatures simultaneously [51]. Furthermore, while TAMs from patients’ samples all exhibited an M2 signature, many TAMs also exhibited a strong M1 signature, termed ‘M1^hot^ TAMS’. These M1^hot^ TAMs were associated with a strong CD8^+^ T_RM_ tumour-infiltrate and better survival outcomes.

The well-documented pro-tumoral activity of TAMs is strongly dependent on exposure to tumour-derived factors, including IL-6, M-CSF, ROS and lactic acid, during macrophage development [52]. TAMs can promote the growth and survival of cancer cells and suppress the anti-tumour immune response through expression of arginase 1 (ARG1), inducible nitric oxide synthase (iNOS), IL-10, transforming growth factor β (TGFβ), and indoleamine 2,3 dioxygenase (IDO) [53]. For example, iNOS is a key enzyme in driving production of nitric oxide (NO) in the TME. NO has been classically recognised as a myeloid-derived immunosuppressive molecule that inhibits anti-tumour T cell survival and function. Yet, Klug et al. demonstrated that iNOS expression by myeloid cells leads to enhanced recruitment of adoptively transferred T cells and was implicated in promoting a T cell response [54]. In addition, high expression of ARG1 is associated with greater histological malignancy and a worse clinical prognosis [55]. However, Vogelpoel et al. showed that ARG1+ M2-like macrophages also produce significant amounts of pro-inflammatory cytokines including TNFα, IL-6, IL-8 upon stimulation [56]. Recent work has helped to demonstrate the role of TAMs in the TME is much more nuanced and context-dependent than originally believed. Future work, particularly multi-omic approaches, will inform on the balance of pro and anti-inflammatory signals necessary to develop improved immunotherapies.

### 3.4. Dendritic Cells

Dendritic cells are recognised as the bridge between innate and adaptive immunity. They function as professional antigen presenting cells to activate antigen-specific CD4^+^ and CD8^+^ T cell responses. Furthermore, they are key producers of pro-inflammatory cytokines such as type I IFN, IL-12 and IL-15. Consequently, they are attractive targets for immunotherapy; targeting DCs in combination with other cancer treatments, such as radiation [57,58], checkpoint blockade [57] or adoptive cell therapy [59], is an effective method of inducing epitope spreading. The phenomenon of epitope spreading describes the induction of T cell immunity against additional cancer antigens secondary to the dominant epitope response. For effective T cell responses to be induced, appropriate co-stimulation signals are required. In the absence of these signals, DCs may promote tumour progression due to the induction of tolerance. This could explain why despite being associated with response to checkpoint blockade in mice [57] and humans [60], cDC1s are also present in tumours that do not respond to checkpoint blockade [61].

The TME is home to several soluble factors that may promote a tolerogenic or immunosuppressive DC phenotype; these include IL-10, IL-6, VEGF, CSF-1, β-catenin, TGF-β and PGE2 [62]. VEGF [63], β-catenin [64] and PGE2 [65,66] production by tumour cells and IL-10 secretion by tumour-associated macrophages [67] have been shown to inhibit DC function and/or recruitment. Furthermore, DCs themselves may produce immunosuppressive cytokines such as IL-6 and IL-10 in response to signals within the TME [68]. Metabolism also plays a key role in the functioning of DCs within the TME. Tumour-derived lactic acid [69] and the accumulation of lipids [70,71] have both been shown to cause DC dysfunction and impaired anti-cancer responses. In addition, upregulation of the enzyme IDO by tumour-associated DCs and the subsequent metabolism of tryptophan, an essential amino acid for effector T cells, can promote Treg differentiation [72]. Clearly understanding the interactions between DCs and the local TME is key to unleashing their anti-tumour potential.

The importance of the cDC1 subset in generating anti-cancer CD8^+^ T cell immunity, and methods of targeting them, have been reviewed extensively elsewhere [73]. Many of these cDC1-targeting strategies have been approved for use in the clinic, such as DC vaccination (NCT00065442; NCT00769704) and the use of adjuvants to promote DC development and/or maturation (NCT01188096; NCT00006249; NCT01465139). Interestingly, cDC2s comprise a larger proportion of the DC population within the TME compared to cDC1 [74,75] and yet receive far less attention. In the steady state, cDC2 are responsible for priming CD4^+^ T helper cell responses. Under inflammatory conditions such as cancer, however, cDC2 cells can transition into an inflammatory cDC2 (inf-cDC2) phenotype characterised by a hybrid gene signature shared with cDC1s and monocyte-derived cells [76]. Furthermore, inf-cDC2 display the cDC1-resricted function of cross-presentation, thus demonstrating an ability to promote both CD4^+^ and CD8^+^ T cell immunity [76]. Already enriched in the TME, targeting these cells may prove an effective method of inducing anti-tumour responses. Conversely, a novel immunoregulatory signature has recently been demonstrated to be upregulated in mature DCs upon uptake of tumour antigens [61]. This “mregDC” program impairs cDC1 function in both human and mouse cancers but can be partially restored using IL-4 signalling blocking antibodies [61]. Incorporating recent advances in our understanding of DC phenotype and functioning in inflammatory states will likely be crucial for the future of DC-targeting immunotherapies.

### 3.5. Myeloid-Derived Suppressor Cells

Myeloid-derived suppressor cells (MDSC) are an immature population of myeloid cells whose function is to reduce inflammation [77]. Chronic inflammation and in turn cancer are characterised by the continuous release of signals and cytokines that induce MDSC [78]. MDSC are divided into two populations, based on their phenotype and morphology: (i) monocytic-MDSCs (M-MDSC) and (ii) polymorphonuclear MDSC (PMN-MDSC) [79]. The discrimination between PMN-MDSCs and neutrophil subpopulations is still subject to debate, with PMN-MDSCs within the TME sometimes referred to as pro-tumoral/anti-inflammatory neutrophils. Interestingly, studies have suggested that tumour-associated neutrophils possess both anti-tumour and pro-tumour properties [80]. Like TAMS, MDSCs use a broad range of suppressive molecules to inhibit antitumor activity, including ARG1, iNOS, IDO, ROS, TGFβ and IL-10 [81]. We recently reviewed the dichotomous role these classically immune-suppressive molecules can play, focusing on both pro- and anti-tumoral effects in the TME [82]. In addition, it has been reported that MDSC can exert immunosuppressive effects via upregulation of PD-L1 [83], expression of the death receptor CD95 to induce T cell apoptosis [84] and production of MMP, which aid in tumour cell extravasation and migration [85].

Importantly, an inverse association between MDSC numbers and clinical response to radio-, chemo-, and immunotherapy have been reported [81]. Studies have recently demonstrated that treatment with ipilimumab (anti-CTLA4) in melanoma patients decreased numbers of both M- and PMN-MDSC and this correlated with favourable therapeutic effects [86,87,88]. As such, targeting MDSCs has become an increasingly popular approach to increase the efficacy of current treatments. Early studies have shown that all-*trans* retinoic acid (ATRA) promoted the differentiation of M-MDSCs into macrophages and DCs and eliminated PMN-MDSCs [89,90]. Furthermore, treatment with IL-2 and anti-CD40 antibody sensitised MDSCs to Fas-mediated apoptosis in multiple murine tumour models [91]. More recently, melanoma patients were treated with ATRA and anti-CTLA4 and this was shown to reduce circulating MDSCs and expression of immunosuppressive genes [92]. In addition, depletion of MDSC levels by chemotherapy improved the efficacy of cancer vaccines in cervical cancer [93]. Therefore, inhibition of MDSC to reduce their immunosuppressive effects in the TME is an attractive target for cancer therapy, with preclinical and clinical data demonstrating promising results to date.

### 3.6. Innate Lymphoid Cells

Innate lymphoid cells (ILCs) are lymphocytes characterised by their lack of antigen-specific receptors. ILCs mirror T cells in their expression of master regulator transcription factors and cytokine production. ILC1s possess Th1 characteristics that include (i) pro-inflammatory and anti-tumour functions, (ii) expression of the master regulatory transcription factor TBET and (iii) production of IFNγ upon activation [94]. Recombinant IFNγ is known to have anti-proliferative, anti-angiogenic and pro-apoptotic effects against cancer cells. Its immunomodulatory functions include the ability to upregulate MHC expression to enhance presentation of antigens by APCs and direct priming by cancer cells [95]. Indeed, the increased immunogenicity of tumour cells allows for elimination by cytotoxic lymphocytes, which are recruited to the tumour by IFNγ-induced chemokine signalling. IFNγ can play a peculiar role specifically because of its very early release by ILC1s. It has been shown to drive recruitment of MDSCs and induce release of IDO, both of which suppress T cell proliferation and favour development of T_regs_ [96].

Unlike ILC1s, ILC2s share similarities with their Th2 counterparts, driven by master regulatory transcription factor GATA3, and produce type 2 cytokines [97]. It is for these reasons they are viewed as anti-inflammatory and pro-tumoral cells. This viewpoint has been challenged by several studies demonstrating the importance of ILC2s in anti-tumour immunity. ILC2s produce type 2 cytokines, primarily IL-5, IL-9 and IL-13, which have been attributed to promoting tumour progression [98]. However, IL-5 drives expansion of eosinophils, the infiltration of which has been demonstrated to improve prognosis in several cancers [99]. Recently, Jacquelot et al. demonstrated that ILC2-derived GM-CSF drove eosinophil recruitment in melanoma tumours enhancing antitumor immunity [100]. Furthermore, they showed that ILC2s expressed PD-1 and administration of IL-33, which drives ILC2 activation, in conjunction with PD-1 blockade enhanced antitumor immunity. Critically they showed a strong correlation between tumour-infiltrating ILC2, eosinophils and improved survival in melanoma patients using gene signature mapping on the TCGA database. The same group also demonstrated a similar pattern in colorectal cancer (CRC) patients and enhanced tumour burden in ILC2-deficient mice compared to wildtype littermates in a CRC model [101]. In summary, ILC2s are associated with both tumour progression and modulating anti-tumour immunity. Understanding what drives these differing outcomes may allow manipulation of the TME to improve treatment responses.

The lesser known ILC3s express RORyt and produce IL-17 and IL-22, analogous to Th17 cells. In mice ILC3s can be further subdivided into NKp46-expressing natural cytotoxicity receptor positive ILCs (NCR^+^ ILC3) and CCR6-expressing lymphoid tissue-induced like ILCs (LTi-like ILC3). NCR^+^ILC3 had a positive correlation with TLS in both NSCLC [102] and CRC [103] and was associated with earlier stages of disease. Accumulating evidence has demonstrated that TLS are important in inhibition of tumour metastasis and has favourable prognosis in multiple cancers [39,40,41]. Furthermore, in a B16 mouse melanoma model LTi-like NCR^+^ILC3s induced ICAM and VCAM leading to increased leukocyte invasion and tumour suppression [104]. Clearly ILCs are a diverse group of cells that play a complex role within the TME, with the potential to be targeted for cancer immunotherapies.

### 3.7. Natural Killer Cells

Natural killer (NK) cells are ILCs pivotal in the early immune response against infection and cancer. NK cells are cytotoxic cells tightly regulated by the balance of activating and inhibitory receptors which bind to MHC-I on target cells, including killer cell immunoglobulin-like receptors [105]. NK cells directly and indirectly kill target cells by exocytosis of cytotoxic granules, expression of FASL and TRAIL, and secretion of cytokines, growth factors and chemokines which shape the innate and adaptive immune response [106]. NK cells can kill tumour cells without prior sensitisation and are critical in inhibiting the initial outgrowth of tumours. Moreover, in a recent systematic review, infiltration of NK cells is associated with improved OS in solid tumours [107].

The TME impedes NK cells activation via multiple factors including hypoxia and soluble factors such as TGF-β, IDO, and PGE2 [105]. TGF-β signalling in NK cells drove their conversion into intermediate-ILC1s (iILC1s) and ILC1s in the TME [108]. Data suggest ILC1-like cells either promote or inhibit tumorigenesis, depending on their phenotype and environmental cues. Indeed, using transgenic mice in which NK cells were hyper-responsive to TGF-β, Gao et al. identified NK cells that instead resembled ILC1s [108]. This phenotypic switch was functionally relevant, as the resulting iILC1s were unable to control tumour burden or viral load in several mouse models [108].

Adoptive transfer of genetically modified NK cells, checkpoint inhibitors and antibodies targeting NK cells are promising immunotherapeutic strategies to eliminate cancer. For example, expression of CD16 on NK cells render them strong mediators of antibody-dependent cellular cytotoxicity (ADCC). Exploiting this, multiple monoclonal antibodies have been developed, including rituximab (anti-CD20), cetuximab (anti-EGFR) and trastuzumab (anti-HER2), and are now standard of care for various cancers [109]. CAR-NKs are becoming increasingly popular, with current clinical trials testing their efficacy in a variety of cancers. Liu et al. generated CAR-NK cells retrovirally transduced to express anti-CD19 CAR, IL-15 and an inducible caspase-9 suicide switch enabling abolition of the cells in vivo [110]. In patients with CD19^+^ B cell lymphoma or CLL, this CAR-NK cell product was associated with complete remission in 7 of 11 patients, without any major adverse effects [111]. Multiple phase I/II clinical trials are currently underway utilising CAR-NKs in solid tumours (NCT02839954, NCT03941457, NCT03940820); no results have been reported to date.

As with T cells, PD-1 expression on NK cells is associated with a reduction in NK cell activity. While PD-1 blockade can unleash T cells against PD-L1-expressing tumours, loss of MHC-I on the tumour surface impacts efficacy of ICB. The success of PD-1/PD-L1 blockade in mice bearing PD-L1^+^ MHC-I^−^ tumours has demonstrated the importance of NK cells [112]. Furthermore, the responsiveness of PD-L1^−^ tumours to anti-PD-L1 therapy has been attributed to PD-L1^+^ NK cells [113]. Importantly, clinical trials have demonstrated that the combination of monalizumab (anti-NKG2A) and durvalumab (anti-PD-L1) were well tolerated in patients with advanced solid tumours (NCT02671435) [114].

### 3.8. B Cells

B cells can account for up to one quarter of all cells within a tumour [115] and approximately one third of cells in tumour-draining lymph nodes [116], highlighting the importance of investigating this cell type. Both B cells and their mature plasma cell counterparts can support anti-tumour immune responses through several mechanisms. Plasma cells secrete tumour-specific IgG1 antibodies that mediate ADCC and phagocytosis of tumour cells. B cells have been shown to promote anti-tumour immunity through the release of inflammatory cytokines, such as IFNγ and IL-12, and directly attack tumour cells via production of granzyme B and TRAIL in hepatocellular carcinoma [117]. In addition, B cells can act as APCs, presenting tumour-associated antigens directly to T cells via B cell receptors or indirectly via antibodies that support the uptake of tumour antigens by TAMs and DCs [118]. Interestingly, B cells express targets of ICB [115], indicating their potential to mediate anti-tumour responses associated with this revolutionary therapy.

Despite their anti-tumour potential, B cells and plasma cells can also promote tumour growth. They release immunomodulatory cytokines, including IL-10, IL-35 and TGFβ, promote immunosuppressive myeloid cells and T_reg_ development, and suppress effector T cells [119]. During an anti-tumour response, B cells produce ineffective antibodies, which form immune complexes and in turn promote chronic inflammation and development of MDSCs [115,119]. In line with these divergent functions, analysis of publicly available RNA-seq data from TCGA revealed that while high expression of B cell and plasma cell gene signatures correlated with improved OS in melanoma, lung adenocarcinoma, PDAC, and head and neck squamous cell carcinoma, whereas poor outcomes were seen in glioblastoma and clear cell renal cell carcinoma [119].

Further insight into the heterogeneity of B cells in the TME comes from recent work in NSCLC. Two classes of tumour-infiltrating B cells with distinct gene expression signatures were identified via scRNAseq: naïve-like B cells and plasma-like B cells. Higher infiltration levels of naïve-like B cells correlated with a better overall survival and relapse-free survival [120]. In addition, IgG^high^ B cells produce immunoglobulins that inhibited cell growth in the early stage of NSCLC but could promote cell growth in advanced stages [120]. Griss et al. analysed the effect of B cells in ICB in human melanoma [121]. Depletion of CD20^+^ B cells decreased overall inflammation, tumour infiltration by CD8^+^ T cells and macrophages, and reduced the tumour-induced plasmablast-like B cell population (TIPB) signature. TIPBs co-expressed immune-stimulatory and -inhibitory cytokines and cell-surface receptors and played a crucial role in sustaining tumour inflammation and recruitment of CD8^+^ T cells. Furthermore, depletion of TIPB cells in the TME decreased overall inflammation and immune cell numbers. Vice versa, the frequency of TIPB cell in pre-therapy samples correlated with improved response and patient survival to ICB. These recent advances in B cell heterogeneity understanding highlight the value of advanced sequencing techniques in informing the future of immunotherapy strategies.

### 3.9. T Cells

CD8^+^ T cells are undoubtably critical in generating anti-tumour responses and considered the principal effector cell in immunotherapy. CD8^+^ T cells are well known for the ability to directly kill both pathogens and neoplastic cells. As such, many immunotherapies aim to induce or reinvigorate CD8^+^ T cell function. Interestingly, cytotoxic T cell immunity in response to chronic infections and tumours is maintained by a specialised population of CD8^+^ T cells that exhibit hallmarks of both exhaustion and memory. These cells give rise to a terminally differentiated exhausted effector cell population which contributes to control of chronic viral infection and tumours [122,123]. Importantly, recent work suggests that precursor exhausted T (T_PEX_) cells are responsible for the enhanced proliferation after ICB and generate the pool of effector T cells [124,125], and are also critical in mediating the response to ACT protocols [126]. Furthermore, increased T_PEX_ cell frequencies have been linked to increased patient survival and improved outcomes in response to therapy [127,128]. The transcription factor TCF1 is central to both conventional memory T cells and T_PEX_ [122,123]. Indeed, expression of TCF1 during the effector stage of T cell differentiation was linked to development of memory T cells in both chronic and acute infection [129]. T cell exhaustion protects T_PEX_ from undergoing differentiation during periods of high and continued antigenic load [130]. This allows their preservation during ongoing infections while simultaneously reducing the risk of immune-mediated collateral damage. Therefore, a deeper understanding of the heterogeneity and relationship between T_PEX_ and exhausted T cells will be critical in development of improved ACT protocols to increase the persistence and durability of transferred cells.

Another population of CD8^+^ T cells critical to anti-tumour immunity are CD103^+^ tissue-resident memory T (T_RM_) cells. T_RM_ reside in the periphery within epithelial tissues and do not recirculate in the blood [131]. A series of studies have shown that T_RM_ cells accumulate in solid tumours, particularly of epithelial origin, and are associated with enhanced anti-cancer immunity in patients [132,133,134,135,136]. Interestingly, TGF-β plays a pivotal role in the formation and maintenance of T_RM_ cells [137]. It has been shown that targeting the TGF-β pathway inhibits tumour growth by promoting anti-tumour immunity associated with increased CD8^+^ T-cell numbers [138]. However, the consequences of depleting TGF-β on T_RM_ cells, which are dependent on TGF-β, has not been assessed. Critically, we have previously shown that T_RM_ are important drivers of immune equilibrium and can control melanoma growth in vivo [139]. Spontaneous disease control in mice was correlated with the generation of tumour-specific T_RM_ cells, where mice remained free of macroscopic lesions long after transplantation of melanoma cells. In addition, T_RM_ have been shown to express a wide range of checkpoint markers, including CTLA-4, TIM3 and PD-1 [140], suggesting the use of ICB could reinvigorate the anti-tumour potential of these cells. Such pre-clinical work and the strong correlation between survival and T_RM_ in clinical data provide a persuasive argument for exploring T_RM_ cells as targets of immunotherapies and highlight the dual role of TGF-β in the TME.

CD4^+^ T cells are polyfunctional cells with a diverse repertoire of effector functions and considerable phenotypic plasticity. It is clear that CD4^+^ T cells are critical effectors in anti-tumour immunity as highlighted by a recent paper by Brentville et al., demonstrating the critical role of CD4^+^ T cells in mediating anti-cancer response to peptide vaccines [141]. In addition, multiple groups have shown the efficacy of CD4^+^ T cells in ACT protocols [142,143,144,145]. Several subsets of CD4^+^ T cells have been described, including Th1, Th2, Th9 and Th17 cells, yet it is unclear which subset is most efficient for ACT. Work has largely focused on the potential of IFNγ-producing Th1 cells [146,147], with all clinical trials to date based on Th1 cell transfer [148,149]. However, other Th subsets may be equally or more efficient as immunotherapies. For example, a preclinical study demonstrated that adoptively transferred Th17 cells were more potent than Th1 cells against established B16 murine melanomas [150]. Critically, Th2 cells, which secrete IL-4, IL-5, and IL-13, have been largely dismissed as they are generally considered to be anti-inflammatory and pro-tumoral [147,151,152]. However, a few early studies have indicated that transfer of Th2 cells may be efficient at eradicating cancer [153,154,155]. A more recent study by Lorvik et al. demonstrated that adoptive transfer of Th2 cells induced a strong type-II inflammatory response within the TME and massive infiltration of macrophages [156]. Moreover, there were increased levels of both pro-inflammatory (IL-1α, IL1-β, TNFα) and Th2-associated (IL-4, IL-5, IL-13) cytokines within the TME. Th2 cytokines induced the expression of classical ‘M2 macrophages’ markers, arginase and CD206 [46], which have often been associated with tumour progression [55]. However, this study clearly demonstrated that arginase-producing ‘M2 macrophages’ were key participants in tumour eradication, in concert with tumour-specific Th2 cells. Furthermore, Chen et al. recently determined, through network analysis, that long-term persistence of CAR-T cells in patients with B cell malignancies was associated with higher expression of Th2 associated transcription factors, including BACH2, FOX2 and GATA3, than those patients with low persistence of the CAR-T cells [8]. Clearly these studies demonstrate the important antitumoral role Th2 cells can play in the TME while simultaneously also being associated with pro-tumoral functions. Significantly, deciphering the subtleties within the Th2 compartment may provide therapeutic targets to augment the TME to improve patient response to immunotherapies.

T_regs_ are a major subset of CD4^+^ T cells, which mediate both tolerogenic and immunosuppressive functions in homoeostatic and inflammatory environments [157,158]. CD4^+^ T_regs_ are most broadly characterised by the expression of transcription factor FoxP3, which acts as a master regulator of immunosuppressive functions [159]. Saito et al. demonstrated that tumour-infiltrating T_regs_ consisted of a FoxP3^hi^ suppressive population and a non-suppressive FoxP3^lo^ population induced by the Th1-polarising cytokine IL-12 which secreted pro-inflammatory cytokines including IFNγ and IL-17 [160]. Interestingly, the authors showed that patients with high infiltration of the non-suppressive FoxP3^lo^ cells had better prognoses than patients with lower infiltration of the same cells. Clearly, even within T cell subsets, there is substantial diversity to consider when targeting these cells for immunotherapy.

## 4. Development of Personalised Immunotherapies Guided by Integrated Omics

**Figure 1:**
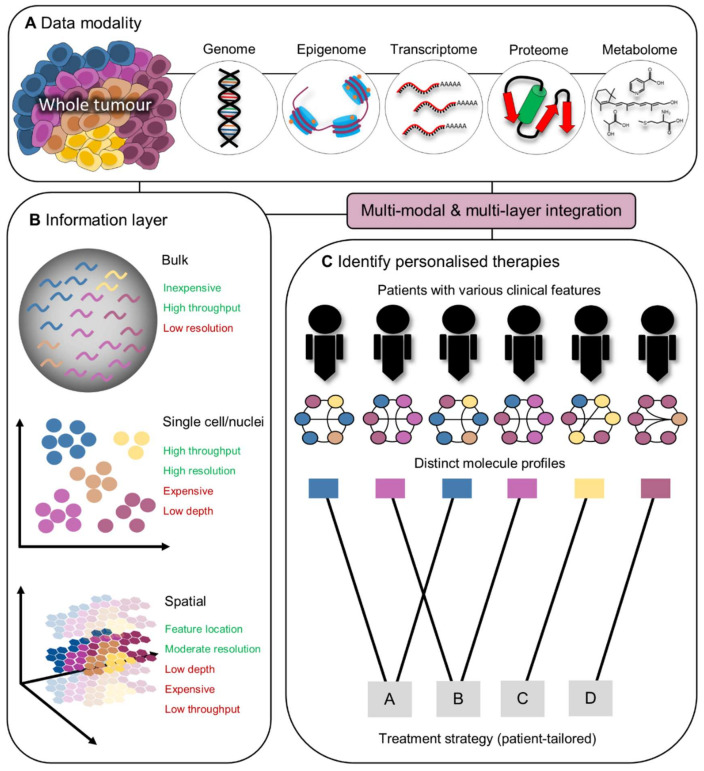
Leveraging the power of omics to guide personalised immunotherapy. (**A**) Whole tumours (and other sample types such as whole blood) contain all the fundamental modalities of information including the genome, epigenome, transcriptome, proteome, and metabolome. (**B**) Modern sequencing technology enables the extraction of information at various layers including bulk, single cell/nuclei and spatial. Each of these methodologies possess inherent disadvantages, however multiple layers can be combined in experiments to mitigate these issues. (**C**) By integrating the various modalities and information layers together, a comprehensive molecular snapshot can be obtained to develop patient-tailored therapies, maximising clinical benefit.

As has been described above, tumours exhibit a remarkable level of complexity that varies significantly across patients. Accordingly, many patients continue to respond poorly to standard of care therapies. It is well established that the immune system requires some degree of ‘fine-tuning’ to elicit effective and long-lasting anti-tumour immunity [82]. With the advancement of high-resolution omics technologies that encompass the global characterisation of DNA, RNA, chromatin accessibility, proteins and metabolites, new personalised treatment strategies can be developed to fine-tune these immune responses and improve individual patient outcomes (Figure 1). This guided approach using single cell genomics has been illustrated by Wang et al., who identified the potential to repurpose tyrosine kinase inhibitors to target the myeloid compartment in treatment for refractory HER2^+^ breast cancers [161]. Similarly, an approach combining bulk and single-cell RNA-seq identified IL-17 as a synergistic pathway that can be targeted to arrest the growth of aggressive gastric cancers [162]. Further studies integrating single-cell protein, epigenetic and transcriptomic information have highlighted novel molecular factors that drive long-term persistence and efficacy of CAR-T cells in vivo [8,163].

Another advantage of single cell sequencing is to leverage the gene expression profiles of individual cells to infer predicted patterns of intercellular communication with a variety of publicly available analysis toolkits [164]. Tumours represent a complex and dynamic ecosystem of cells that are frequently transmitting and receiving signals, and this cross-talk can promote the growth of tumour cells and/or propagate the immunosuppressive TME. For example, the mapping of ligand–receptor signalling paths between tumour stem cells and TAMs highlighted a novel chemoradiotherapy-resistant mechanism driven by immunosuppressive PGE-2/EP-4 signalling [165]. In line with this theme, Zhang et al. defined critical intercellular interactions between myeloid cells and the TME underpinning resistance to myeloid-targeted immunotherapies such as anti-CSF1R and CD40 agonists [166]. Similar cell-to-cell communication analyses in advanced renal cell carcinoma reveal the potential to combine anti-CD47 antagonists with anti-PD-1 therapy to augment macrophage-mediated phagocytosis of tumour cells [167].

Spatial omics represents the newest frontier of high-dimensional analysis by allowing users to map the abundance of proteins, RNA, and metabolites to specific regions of tissue. Although current specifications lack single-cell resolution and remain uneconomical for larger cohort studies, this technology can leverage the strengths of other approaches to gain a more comprehensive snapshot of the TME. This is highlighted by Vathiotis et al., where the combination of spatially resolved protein expression and bulk mRNA from corresponding tumours in melanoma patients could predict clinical outcomes more accurately compared to either variable alone [168]. Furthermore, using an approach combining snRNA-seq, spatial transcriptomics and high parameter IHC, Gouin et al. recently uncovered communities of Cadherin 12+ epithelial cells and exhausted CD8^+^ T cells in tumours from bladder cancer patients. Strikingly, although tumours enriched in these cellular niches were more resistant to neoadjuvant chemotherapy and surgery, they were associated with superior outcomes following PD-L1 blockade [169]. These combined technologies also provide novel mechanistic insights underpinning durable responses to therapy. For instance, improved outcomes following ICB in melanoma patients correlated with the formation of CD8+CD20+ TLS [170], while reduced tumour burden in melanoma-bearing mice following anti-TGFβ relies on rendering tumours more permissible to T cell attack by remodelling the tumour stroma [171]. Moreover, liver metastases from CRC patients that respond to neoadjuvant chemotherapy significantly alter the spatiotemporal landscape of MRC1+CCL18+ M2 macrophages that are otherwise absent in non-responders, highlighting the identification of patient subsets that may benefit from combination immunotherapies that selectively target these tumour-promoting immune cells [172]. Spatial metabolomics also unveils novel fatty acids that may be an important correlate of tumour-infiltrating lymphocyte (TIL) content in tumours from CRC patients [173]. Moreover, the use of mass spectrometry imaging detected a novel association between neutrophil defensins in NSCLC and better clinical responses to anti-PD-L1 therapy [174]. The embracing of this integrative approach has also culminated in the construction of comprehensive cancer atlas’ by combining single cell and spatial information, leading to the identification of ‘ecotypes’, which define tumour subtypes with unique cellular contextures and clinical outcomes [175]. Collectively, these studies demonstrate the power of leveraging multi-omics to unmask the mechanisms of anti-tumour immunity and develop novel, personalised immunotherapeutic strategies.

Despite the unparalleled resolution of single cell and spatial technology, cost and throughput issues hinder their ability to be deployed to the clinic at the present time. However, bulk sequencing being significantly more cost effective (at the expense of resolution) may have a lower barrier-to-entry into the precision medicine field. Demonstrating the feasability of this concept, Xia et al. applied a deep learning computational approach to integrate bulk mRNA, protein and microRNA profiles of over 60 tumour cell lines to predict responses against various drug combinations [176]. With the field rapidly evolving, we anticipate that new omics technologies, particularly those profiling at single cell and spatial resolution will play a more dominant role in personalised immunotherapy in the years to come.

## 5. Conclusions

**Figure 2:**
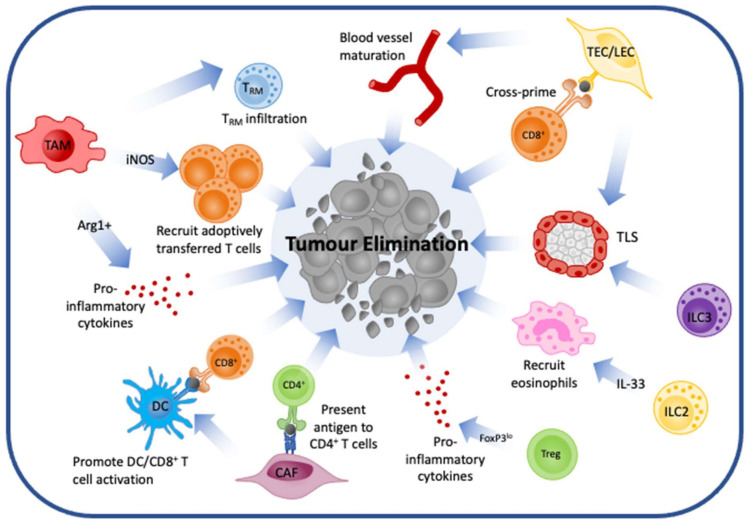
The anti-tumoral roles of classically pro-tumoral cells in the tumour microenvironment. TAMs, TECs, CAFs, ILCs and T_regs_ are often classified as pro-tumoral, however growing evidence supports anti-tumoral roles for these cells which aid in the elimination of cancer. These include the secretion of pro-inflammatory cytokines and the activation of additional anti-tumoral cells, in particular effector CD8^+^ T cells. TAM: tumour associated macrophage, TEC/LEC: tumour endothelial cell/lymphoid endothelial cell, CAF: cancer associated fibroblast, T_RM_: tissue resident memory T cell, DC: dendritic cell, ILC: innate lymphoid cell, TLS: tertiary lymphoid structure.

Solid tumours are a complex arrangement of cells, vessels, and soluble factors with both pro- and anti-tumoral activity, which vary greatly both within and across cancer types and patients. As such, we see great diversity in patient response rates to immunotherapies. To stratify patients to determine which may respond to immunotherapies we rely upon categorising tumours as ‘hot’ or ‘cold. Unfortunately, this oversimplification of the nuanced and context-dependent nature of the TME leaves gaps in our understanding of why some patients do not respond to immunotherapy and other do despite having ‘hot’ or ‘cold’ tumours, respectively. Current work now appreciates the abundant diversity within cell populations in the TME and the impact this has upon response to immunotherapies. In particular, certain cells have been typically overlooked and classified as ‘pro-tumoral’, dismissing the various functions these cells have in inducing an immune response that can aid in the elimination of cancers (Figure 2). This review has highlighted key cells within the TME and the diverse and heterogeneous roles they can play. Further we discuss how these different cells are being targeted to improve current immunotherapeutic strategies. However, future work will need to further unravel the complexities of the TME to understand what drives the differing functions within cells and how these may be targeted. Critically, the massive leap forward in multi-omic technologies is now providing opportunities to unmask the mechanisms of anti-tumour immunity at exquisite detail. This deeper understanding of an individual patient’s tumour may allow stratification of patients for personalised treatment modalities. This emphasises that a comprehensive understanding of the TME is critical in developing the next breakthroughs in immunotherapy.
